# ‘Film is psychosis’: filmmakers with lived experience

**DOI:** 10.1192/j.eurpsy.2025.208

**Published:** 2025-08-26

**Authors:** S. Anderson

**Affiliations:** Psychotherapy Science, Sigmund Freud Private University Vienna, Vienna, Austria

## Abstract

**Abstract:**

The presentation juxtaposes the practice of psychiatry with the creative processes involved in filmmaking. It proposes a correlation between the representation of reality in film, and the processing of visual information. It is informed by autobiographical case study films written and directed by individuals with experience of psychosis. In-depth analysis of film conventions employed by filmmakers with experience of psychosis indicate the potential benefits of further research into collaboration with, and contributions from, those filmmakers.

**Disclosure of Interest:**

None Declared

